# Epidemiological trends and projections to 2035 of hepatitis B burden in China, 1990–2021

**DOI:** 10.1371/journal.pone.0330633

**Published:** 2025-09-02

**Authors:** Xinyue Qi, Yongzheng Hu, Wei Jiang

**Affiliations:** Department of Nephrology, The Affiliated Hospital of Qingdao University, Qingdao, Shandong, China; National Center for Chronic and Noncommunicable Disease Control and Prevention, Chinese Center for Disease Control and Prevention, CHINA

## Abstract

**Background:**

Hepatitis B virus (HBV) remains a major cause of liver disease and premature death globally, with China bearing a significant share of the burden. However, long-term trends and future projections of HBV burden in China remain insufficiently described, despite widespread infant vaccination and expanded antiviral therapy.

**Methods:**

We analyzed hepatitis B–related mortality, age-standardized mortality rate (ASMR), disability-adjusted life years (DALYs), and age-standardized DALY rate (ASDR) in China from 1990 to 2021 using data extracted from the GBD 2021 database. Temporal trends were quantified by estimated annual percentage change (EAPC).

**Results:**

Between 1990 and 2021, the number of deaths from hepatitis B-related liver diseases in China increased from 215 thousand to 219 thousand (+1.83%), whereas the ASMR decreased from 23.86 per 100,000 to 10.68 (EAPC: −2.78). The disability-adjusted life years (DALYs) decreased from 7.78 million in 1990 to 6.61 million in 2021 (−15.04%), with the ASDR decreasing from 774 per 100,000–329 (EAPC: −3). Despite significant reductions in HBV mortality and DALYs, the growing burden of HBV-related liver cancer, particularly among older males, remains a critical concern. Targeted early screening and treatment strategies are urgently needed. Projections for 2035 indicate a continued decline in the overall burden of hepatitis B, with the liver cancer burden showing a fluctuating decrease.

**Conclusion:**

Despite progress in HBV prevention and control, the increasing burden of hepatitis B–related liver cancer remains a major public health challenge.

## Introduction

The global burden of hepatitis B virus (HBV) infection continues to pose substantial public health challenges [1], as evidenced by the approximately 257 million chronic carriers worldwide, contributing significantly to the overall hepatitis disease burden [2]. Hepatitis B virus infection can lead to acute hepatitis B, chronic hepatitis B, cirrhosis, and hepatocellular carcinoma (HCC), with different stages of disease progression presenting distinct clinical manifestations and risks [3]. Acute hepatitis B typically occurs within weeks to months following HBV infection and is characterized by a clinical presentation that includes but is not limited to icterus, asthenia, anorexia, and emesis [4]. Chronic hepatitis B is a major contributor to the global burden of liver disease and has the potential to progress to cirrhosis and liver cancer [5]. Chronic carriers may experience liver damage even during the asymptomatic phase, and some patients may develop liver fibrosis due to prolonged inflammation, ultimately progressing to cirrhosis or hepatocellular carcinoma [6].

Among the most serious consequences of persistent HBV infection, HBV-related hepatocellular carcinoma accounts for approximately 50% of all HCC cases with an established viral etiology worldwide [7]. In highly endemic regions such as China, Southeast Asia, and sub-Saharan Africa, this proportion can reach 70% [8–10]. The development of HBV-related HCC typically involves a prolonged process of chronic inflammation, liver fibrosis, and cirrhosis, with mechanisms such as viral integration into the host genome and the carcinogenic effects of the HBV X protein playing key roles [11,12]. Because HCC is often asymptomatic in its early stages, most patients are diagnosed at advanced stages, where treatment options are limited and the 5-year survival rate remains low [13].

In response to this global health challenge, the World Health Organization (WHO) has established an ambitious target to eliminate viral hepatitis as a public health threat by 2030, with specific objectives to attain 90% diagnostic detection rates and 80% therapeutic coverage worldwide [14]. In recent decades, infant hepatitis B vaccination was incorporated into national immunization programs in the United States, Japan, and most European countries, leading to new infection rates below 0.5% [15–18]. These countries also established widespread screening of high-risk populations and ensured timely antiviral therapy, resulting in substantial declines in cirrhosis and HCC [19,20]. In China, the newborn vaccination program began in 1992 and expanded to a national free program by 2002, which reduced HBsAg prevalence from over 10% to around 6% in children.

Current estimates reveal that the prevalence of hepatitis B in China is approximately 75 million cases, constituting nearly one-third of the worldwide chronic HBV infection pool (254 million cases), with a disproportionately high share of global HBV-attributable disability-adjusted life years (DALYs) [21]. Although nucleoside analogs such as entecavir and tenofovir are now covered by national insurance and have lowered treatment costs, adult screening coverage is only around 50%, and antiviral treatment uptake is only approximately one-third nationwide (and under one-fifth in rural areas), far from the WHO’s 80% target [21–23]. Addressing these gaps in screening and treatment delivery is crucial for China to achieve its hepatitis B elimination goals.

China’s national health policy frameworks, including the “Healthy China 2030 Plan” and the 14th Five-Year Plan with the 2035 vision, set clear goals for disease prevention, public health system strengthening, and chronic disease control. The 2030 plan emphasizes expanding healthcare services and reducing major disease incidence, and the 2035 vision explicitly targets a continuation of health gains and further control of chronic diseases [24,25]. These policy milestones provide strong justification for projecting hepatitis B burden through to 2035.

The data for this study were sourced from the Global Burden of Diseases, Injuries, and Risk Factors Study (GBD) 2021, which aims to analyze the epidemiological trends, diagnosis and treatment status, and effectiveness of related policies in HBV infection in China thoroughly, revealing the current status and challenges for HBV management. Although the GBD database cannot capture absolutely comprehensive data—such as detailed regional variations within China—it remains a powerful tool for measuring and evaluating disease epidemiology at the national level [26]. Through a systematic assessment of national data, we aim to provide scientific evidence for developing more effective prevention and control strategies, supporting the achievement of the global goal of eliminating hepatitis B by 2030.

## Methods

### Ethical approval and consent to participate

This research adhered to the principles of the Declaration of Helsinki and complied with applicable local laws. As the study utilized publicly available data, it was exempted from ethical review by the institutional ethics committee.

### Data collection

This research employed comprehensive health metrics obtained from the 2021 iteration of the Global Burden of Disease (GBD) database, a robust epidemiological resource developed and managed by the Institute for Health Metrics and Evaluation (IHME), University of Washington. The database includes detailed information on a wide range of diseases, injuries, and risk factors worldwide. We used the GBD Results Tool (http://ghdx.healthdata.org/) to obtain specific data related to hepatitis B. This study utilized data from the GBD database on hepatitis B-related liver diseases, including acute HBV infection, chronic HBV infection with or without cirrhotic complications, and HBV-induced hepatocellular carcinoma. To facilitate cross-regional and temporal comparisons, all epidemiological metrics underwent rigorous age-standardization procedures. The GBD 2021 estimates derive from a globally standardized and validated framework developed by IHME, synthesizing tens to hundreds of thousands of data sources—including health surveys, registries, administrative records, and peer-reviewed literature. For most indicators, GBD employs robust modeling tools—Cause of Death Ensemble model (CODEm), Spatiotemporal Gaussian Process Regression (ST-GPR), and DisMod-MR 2.1—to generate estimates by age, sex, location, and year, accompanied by 95% uncertainty intervals. All estimates adhere to Guidelines for Accurate and Transparent Health Estimates Reporting guidelines, ensuring transparency, reproducibility, and comparability across regions and time periods [27]. The GBD data are based on an extensive review of the literature, expert assessments, and statistical modeling, and have undergone strict quality control, making them well suited for epidemiological and public health research, thus providing a solid and reliable data foundation for this study.

To quantify the overall health burden of hepatitis B-related liver diseases, we used disability-adjusted life years (DALYs), which sum years of life lost (YLL) due to premature mortality and years lived with disability (YLD). YLL were calculated by multiplying the number of deaths at each age by standard life expectancy at that age, while YLD were estimated by multiplying incident cases by the average duration of disease and a disability weight reflecting its severity. By combining both fatal and non-fatal health loss into a single metric, DALYs enable standardized comparisons of disease burden across time periods, geographic regions, and population subgroups, and facilitate evaluation of the impact of diagnostic, treatment, and policy interventions [28].

To explore differences between various populations, we first divided the study subjects into four main age groups (<35 years, 35–49 years, 50–69 years, and ≥70 years). For a more detailed analysis of the effects of age, sex, and disease stage, we subdivided age into five-year intervals, such as 0–4 years, 5–9 years, 10–14 years, and so on, up to >95 years. After stratification by both age cohort and sex, comprehensive analyses were performed to assess temporal variations in mortality indices and DALYs related to HBV-induced hepatic diseases.

### Estimated annual percentage change (EAPC) analysis

A rigorous statistical evaluation was performed to analyze mortality trends associated with HBV-related hepatic pathologies from 1990 to 2021, covering overall liver diseases, acute HBV infection, chronic HBV infection with or without cirrhotic complications, and HBV-induced hepatocellular carcinoma. The percentage change in the number of deaths was calculated using the following formula:


$$PercentageChange=\frac{\left(Deaths2021-Deaths1990\right)}{Deaths1990}\times 100\%$$

This metric allows us to directly assess the relative changes in the absolute number of deaths across different disease categories. To quantitatively assess temporal mortality risk patterns, we employed the estimated annual percentage change (EAPC) methodology to analyze age-standardized mortality rate (ASMR) trajectories. Specifically, we first performed a natural logarithmic transformation of the ASMR for each year and constructed a linear regression model:


ln(ASMR)=A+b×(Year)+ε

In the specified model architecture, A corresponds to the intercept parameter, b signifies the regression coefficient characterizing the yearly variation in ln-transformed age-standardized mortality rates (ln(ASMR)), utilizing year as the temporal predictor, and ε accounts for random variability in the model. The EAPC is then calculated using the following formula:


$$EAPC=100\times (e{\mathrm{\backslash nolimits}}^{b}-1)$$

The regression coefficient b is converted into the annual percentage change rate, with the 95% confidence interval for the EAPC calculated using the standard error of b. If the EAPC and its 95% confidence interval are both positive, it indicates an upward trend in the ASMR; if both are negative, it suggests a downward trend in the ASMR [29].

In addition, age-standardized disability-adjusted life years (ASDR) metrics and their corresponding EAPC values were computed for HBV-related hepatic disorders to evaluate temporal patterns in disease burden evolution. All the statistical analyses were conducted utilizing R statistical software (version 4.2.3), employing two-tailed hypothesis testing with a predetermined significance threshold of P < 0.05.

### Decomposition analysis

Decomposition analysis is widely used in epidemiology, public health, and health research to assess changes in disease burden, particularly in the GBD study. This investigation utilized decomposition methodology to examine temporal variations in DALYs attributable to HBV-related pathologies in both Chinese and global contexts from 1990 to 2021, while identifying key contributing determinants [30]. The analysis specifically focused on differences stratified by sex (male, female) and disease stage (acute hepatitis B, chronic liver diseases including cirrhosis, and liver cancer) and decomposed the changes in disease burden into three main components: age structure, epidemiological changes, and population size.

### Bayesian age-period-cohort (BAPC) model prediction

In this study, we employed the Bayesian Age-Period-Cohort (BAPC) model to project the future burden of hepatitis B-related diseases, including acute hepatitis B, chronic hepatitis B with cirrhosis, and HBV-associated liver cancer. The BAPC model effectively captures the effects of age, calendar period, and birth cohort on disease outcomes within a Bayesian framework, enabling flexible modeling of temporal trends and cohort effects over time [31]. Compared with other forecasting models, BAPC offers improved flexibility in capturing temporal patterns and better accommodates sparse or noisy epidemiological data.

The model incorporates a second-order random walk prior to smooth age, period, and cohort effects, balancing fit and complexity to improve prediction accuracy. Prior distributions for the model parameters were specified as Gaussian distributions with mean zero and precision parameters reflecting our assumptions about smoothness and variability. Hyper-parameters controlling these precisions were assigned non-informative Gamma priors, allowing the data to guide the extent of smoothing.

An important methodological advantage of the BAPC model is its use of Integrated Nested Laplace Approximation (INLA) to approximate posterior distributions efficiently. INLA provides accurate estimates while avoiding the computational burden and convergence issues commonly associated with Markov Chain Monte Carlo (MCMC) methods.

We implemented the BAPC analysis using the “BAPC” R package (version 0.0.36), integrating hepatitis B burden data from the Global Burden of Disease (GBD) 2021 study with population projections from the Institute for Health Metrics and Evaluation (IHME). This approach allows robust long-term forecasting of disease burden trends and supports policy planning.

## Results

### Trends in China

**Fig 1:**
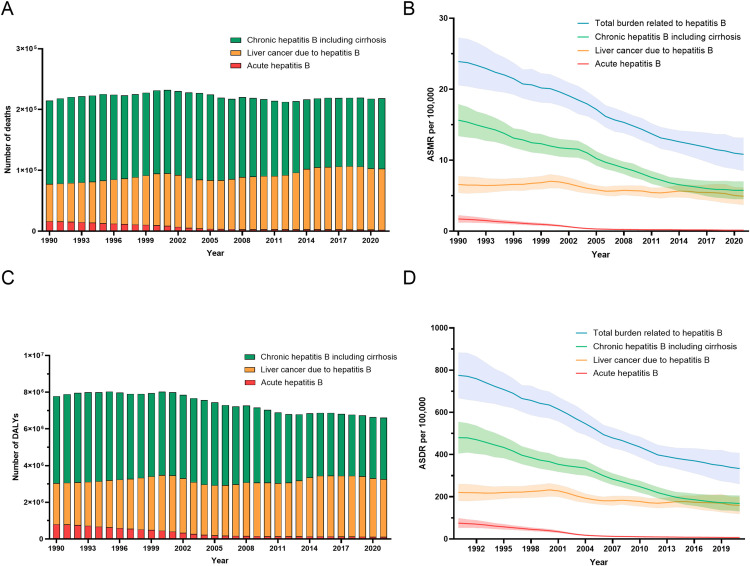
Contribution of acute hepatitis B, cirrhosis and other chronic diseases due to hepatitis B and liver cancer due to hepatitis B to deaths and DALYs of total liver diseases due to hepatitis B in China from 1990 to 2021. **(A)** Mortality counts across the three stages of liver disease. **(B)** ASMRs across the three stages of liver disease. **(C)** DALYs across the three stages of liver disease. **(D)** ASDRs across the three stages of liver disease. The solid line represents the ASMR or ASDR. The shaded bands indicate the 95% uncertainty interval (95% UI). DALYs: disability-adjusted life-years; ASMR: age-standardized mortality rate; ASDR: age-standardized disability-adjusted life-year rate.

The mortality and disease burden rates of hepatitis B-related liver diseases have shown an overall declining trend since 1990. In China, the number of deaths caused by hepatitis B-related liver diseases increased from 215 thousand in 1990–219 thousand in 2021, a slight increase of 1.83%, even as the age-standardized mortality rate (ASMR) decreased significantly from 23.86 per 100,000 population in 1990 to 10.68 per 100,000 population in 2021, with an estimated annual percentage change (EAPC) of −2.78, indicating a continuous downward trend (Table 1, Fig 1B).

**Table 1:** Deaths and DALYs from hepatitis B-related liver diseases in 1990 and 2021 and their change trends from 1990 to 2021.

	Both	Male	Female	Acute hepatitis B	Cirrhosis and other chronic liver diseases	Liver cancer
1990						
Deaths (95% UI)	2.15 (1.85-2.45)	1.54 (1.28-1.85)	0.61 (0.49-0.73)	0.16 (0.11-0.22)	1.37 (1.16-1.58)	0.61 (0.51-0.73)
DALYs (95% UI)	77.82 (67.33-89.13)	57.89 (48.65-69.48)	19.93 (16.26-23.9)	8.03 (5.56-10.59)	47.43 (39.95-55)	22.36 (18.43-26.63)
ASMR (95% UI)	23.86 (20.56-27.26)	33.72 (28.28-40.24)	14.16 (11.54-16.82)	1.69 (1.21-2.25)	15.64 (13.22-18.06)	6.53 (5.42-7.76)
ASDR (95% UI)	774.27 (667.73-883.76)	1114.06 (938.42-1334.08)	417.47 (340.98-498.27)	74.9 (52.05-98.66)	479.32 (405.53-555.49)	220.05 (181.34-260.91)
2021						
Death (95% UI)	2.19 (1.75-2.72)	1.72 (1.33-2.25)	0.47 (0.36-0.62)	0.024 (0.018-0.032)	1.16 (0.91-1.43)	1 (0.78-1.29)
DALYs (95% UI)	66.11 (52.56-82.4)	54.17 (41.8-71.15)	11.95 (9.13-15.91)	1.14 (0.88-1.47)	33.49 (26.31-41.58)	31.49 (24.43-41.09)
ASMR (95% UI)	10.68 (8.57-13.22)	17.54 (13.69-22.78)	4.31 (3.29-5.77)	0.13 (0.1-0.17)	5.72 (4.52-7.03)	4.83 (3.76-6.19)
ASDR (95% UI)	329.66 (262.66-409.33)	545.82 (424.43-713.21)	114.11 (87.48-151.18)	6.81 (5.27-8.61)	167.03 (131.45-206.94)	155.81 (121.32-201.99)
1990-2021						
Deaths (%)	1.83%	11.91%	−23.58%	−84.94%	−15.45%	63.14%
DALYs(%)	−15.04%	−6.43%	−40.07%	−85.77%	−29.40%	40.81%
EAPC of ASMR (95% CI)	−2.78 (−2.89--2.67)	−2.25 (−2.36--2.14)	−4.12 (−4.27--3.98)	−8.98 (−9.72--8.23)	−3.54 (−3.72--3.36)	−0.97 (−1.16--0.78)
EAPC of ASDR (95% CI)	−3 (−3.11--2.89)	−2.5 (−2.61--2.4)	−4.54 (−4.69--4.4)	−8.52 (−9.21--7.83)	−3.72 (−3.88--3.56)	−1.22 (−1.42--1.03)

Note: Deaths, DALYs, ASMR, and ASDR are all expressed per 100,000 population. EAPC is shown as a percentage without units. Percentage values were calculated based on original GBD data prior to unit conversion; minor discrepancies may occur due to rounding. DALYs: disability-adjusted life-years; ASMR: Age-standardized mortality rate; ASDR: Age-standardized DALY rate; 95% UI = 95% Uncertainty Interval.

In contrast to mortality counts, the disability-adjusted life years (DALYs) attributable to hepatitis B-induced liver diseases were 6.61 million in 2021, representing a 15.04% decrease compared with 7.78 million in 1990. The age-standardized DALY rate (ASDR) for the total burden of liver diseases caused by hepatitis B also declined from 774 per 100,000 population in 1990–329 per 100,000 population in 2021, with an EAPC showing a downward trend (EAPC: −3; 95% UI: −3.11 to −2.89) (Table 1).

### Trends by disease stage

All HBV-related disease burdens showed a decreasing trend, but liver cancer remained relatively stable and accounted for an increasing proportion of the total burden. The aggregate disease burden attributable to HBV-related hepatic pathologies encompasses acute HBV infection, chronic HBV infection with cirrhotic complications, and HBV-induced hepatocellular carcinoma. Notably, in China’s 2021 epidemiological landscape, chronic HBV infection with cirrhosis and HBV-associated liver malignancies collectively accounted for approximately 98% of total hepatitis B-related hepatic mortality. During the 1990−2021 period, the annual mortality rates significantly declined for acute hepatitis B (16 thousand to 2.42 thousand deaths; −84.94%) and chronic hepatitis B with the progression of hepatic fibrosis (137 thousand to 116 thousand deaths; −15.45%). Conversely, HBV-related primary liver malignancies showed a marked increasing trend in terms of mortality (61 thousand to 100 thousand deaths; 63.14% increase) (Fig 1A). In 2021, the ASMR for each disease stage was 0.13 (95% UI: 0.1–0.17) for acute hepatitis B, 5.72 (95% UI: 4.52–7.03) for chronic hepatitis B with cirrhosis, and 4.83 (95% UI: 3.76–6.19) for hepatitis B-attributable liver cancer (Table 1). While the ASMRs for patients with chronic hepatitis B with cirrhosis and acute hepatitis B decreased over time, the ASMR for patients with liver cancer remained relatively stable (Fig 1B).

The changes in DALYs across different disease stages of hepatitis B-related liver diseases followed trends similar to those of mortality (Fig 1C and D). Notably, DALYs due to liver cancer have gradually increased, becoming a significant component of the total burden over time(Fig 1C). Among these stages, chronic hepatitis B with cirrhosis exhibited the greatest decrease in the ASDR (Fig 1D). The EAPC of the ASMR was −8.98 (95% CI: −9.72 to −8.23) for acute hepatitis B, −3.54 (95% CI: −3.72 to −3.36) for chronic hepatitis B with cirrhosis, and −0.97 (95% CI: −1.16 to −0.78) for liver cancer. The EAPC of the ASDR was −8.52 (95% CI: −9.21 to −7.83) for acute hepatitis B, −3.72 (95% CI: −3.88 to −3.56) for chronic hepatitis B with cirrhosis, and −1.22 (95% CI: −1.42 to −1.03), with all disease stages showing a consistent downward trend (Table 1).

### Age and sex distributions

From 1990 to 2021, hepatitis B burden in China declined overall but showed significant sex and age disparities, with males experiencing higher mortality and DALYs, and the greatest burden concentrated in middle-aged and elderly groups. In the Chinese population, HBV-related hepatic mortality demonstrated gender-specific trends from 1990 to 2021: male fatalities increased from 154 thousand to 172 thousand cases (11.91% increase), whereas female mortality decreased from 61 thousand to 47 thousand cases (23.58% reduction). In 2021, the ASMR for males was 17.54 (95% UI: 13.69–22.78), which was significantly greater than that for females at 4.31(95% UI: 3.29–5.77). The ASMR for females showed a more pronounced downward trend than that for males (EAPC: −4.12, 95% CI: −4.27 to −3.98 vs. −2.25, 95% CI: −2.36 to −2.14) (Table 1).

**Fig 2:**
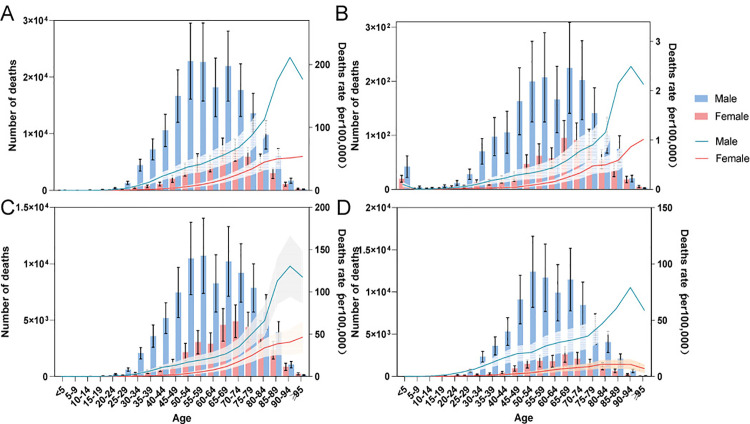
Age patterns by sex of the total number and age-specific death rates in 2021. **(A)** Total burden related to hepatitis B, **(B)** acute hepatitis B, **(C)** chronic hepatitis B including cirrhosis, and **(D)** liver cancer due to hepatitis B.

Notably, in 2021, the mortality rates for all liver disease stages caused by hepatitis B were higher in males than in females across all age subgroups. Male deaths were clustered in the 45- to 79-year-old age group, whereas female deaths were more concentrated in the 65- to 84-year-old age group. Additionally, acute hepatitis B caused notable mortality among children under five years of age, indicating a need for greater attention to this age group (Fig 2). Overall,males bear a greater hepatitis B burden than females do.

**Fig 3:**
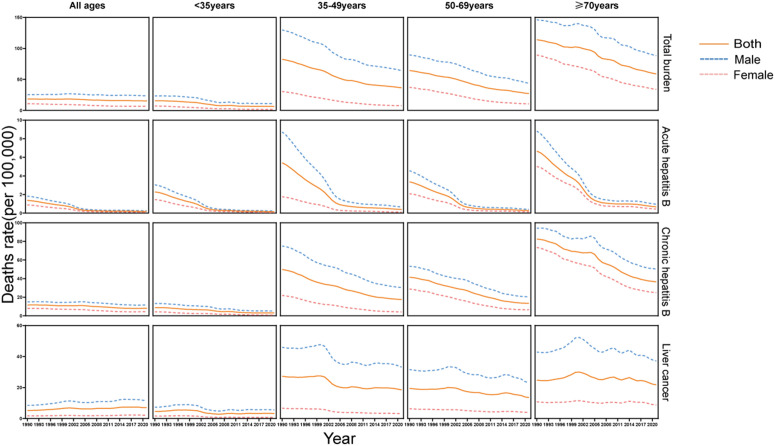
Temporal trends of mortality of the total burden related to hepatitis B and three disease stages for all ages and across four age groups (<35, 35–49, 50–69, and ≥ 70 years) from 1990 to 2021 by sex and age group in China.

Between 1990 and 2021, sex-specific mortality patterns revealed consistently elevated rates among male populations compared with their female counterparts across all disease progression stages. Mortality rates significantly decreased for both acute hepatitis B and chronic hepatitis B. In the case of acute hepatitis B, a rapid decline was initially observed, followed by a more gradual decrease across all age groups. However, the mortality of patients with liver cancer showed no significant decrease, with the rates fluctuating over time. Mortality rates were especially high among those over 70 across all disease stages, highlighting the need for targeted hepatitis B prevention and prognosis efforts among elderly individuals (Fig 3).

From 1990 to 2021, DALYs declined for both sexes, from 5.79 million to 5.42 million for males and from 1.99 million to 1.2 million for females, representing reductions of 6.43% and 40.07%, respectively. The ASDR for males decreased from 1,114.06 to 545.82 (EAPC of DALYs: −2.5, 95% CI: −2.61 to −2.4), and for females, it decreased from 417.47 to 114.11 (EAPC of DALYs: −4.54, 95% CI: −4.69 to −4.4), indicating a more pronounced annual percentage change for females (Table 1).

**Fig 4:**
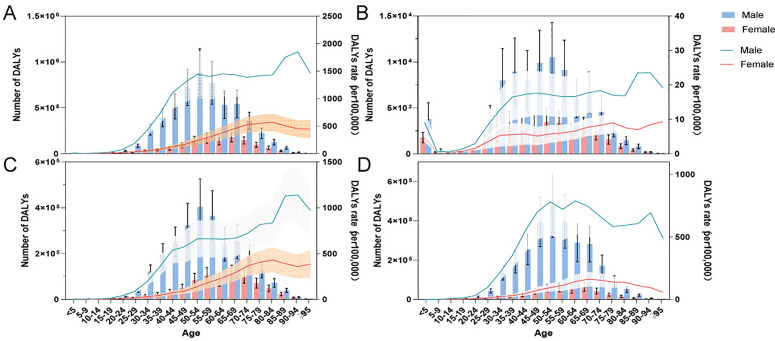
Age patterns by sex of the total number and age-specific DALYs rate. **(A)** Total burden related to hepatitis B; **(B)** acute hepatitis B; **(C)** chronic hepatitis B including cirrhosis; **(D)** liver cancer due to hepatitis B in 2021. DALYs: disability-adjusted life-years.

In 2021, DALYs for males were concentrated primarily in the 45–59 age range, with acute hepatitis B DALYs mostly concentrated between the ages of 30 and 59. For females, DALYs were concentrated among individuals aged 30–59 years, with acute hepatitis B DALYs shifting towards younger individuals (Fig 4). Similarly, trends in DALY rates over time mirrored those of mortality rates (Fig S1).

### Decomposition analysis

**Fig 5:**
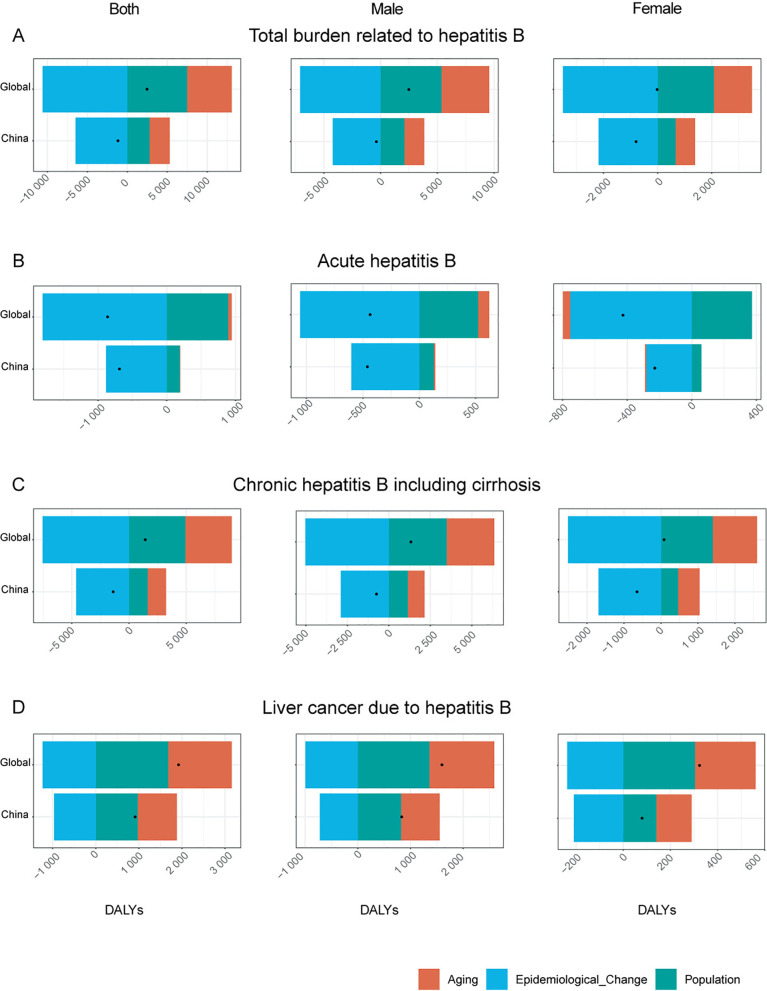
Decomposition analysis of changes in hepatitis B virus (HBV) burden by region (China vs. Global), sex, and disease stage, 1990 to 2021 (in Thousands). **(A)** Total burden related to hepatitis B; **(B)** acute hepatitis B; **(C)** chronic hepatitis B, including cirrhosis; and **(D)** liver cancer due to hepatitis B. The black dots represent the total change contributed by all three components.

Population aging increased hepatitis B burden, but epidemiological improvements reduced DALYs in China. The decomposition analysis revealed the relative contributions of population aging, population growth, and epidemiological changes to the DALYs of hepatitis B infection in China and globally. In contrast with the increasing global burden pattern, China exhibited a reduction in hepatitis B-associated DALYs from 1990 through 2021. Although population growth and aging exacerbated the DALYs, epidemiological changes played a crucial role in reducing the DALYs by 6.48 million, leading to a net reduction of 1.17 million. Stratified by disease progression stage, China presented a reduction in the chronic HBV infection burden (encompassing cirrhotic complications) relative to global patterns. Conversely, HBV-induced hepatocellular carcinoma demonstrated an upward trend in DALYs both in China and worldwide, which is driven primarily by demographic transitions, including population aging and expansion (Fig 5).

### Prediction of DALYs for hepatitis B disease stages in China by 2035

**Fig 6:**
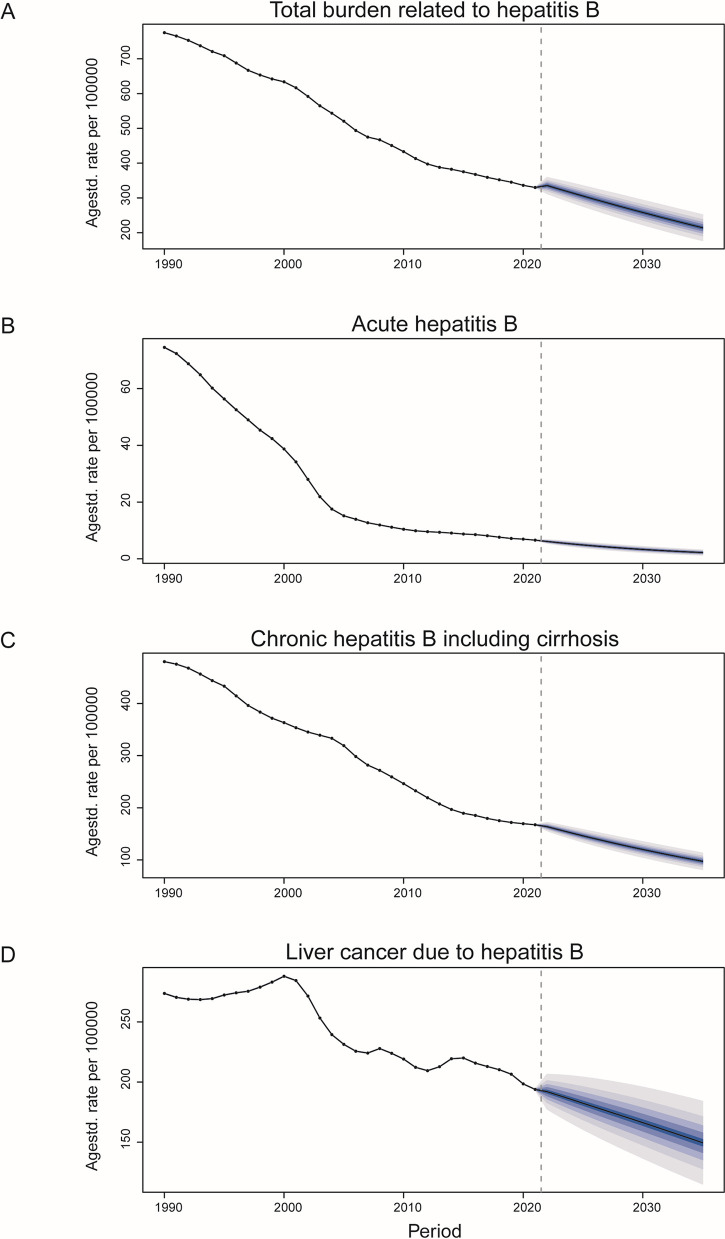
Predictions for the annual age-standardized disability-adjusted life years (ASDR) attributable to hepatitis B virus (HBV) infection in China until 2035 on the basis of the Bayesian age–period–cohort (BAPC) model, stratified by disease stage. **(A)** Total burden related to hepatitis B; **(B)** acute hepatitis B; **(C)** chronic hepatitis B, including cirrhosis; and **(D)** liver cancer due to hepatitis B.

The BAPC model predicts a continued decline in hepatitis B burden in China across all disease stages through 2035, with notable decreases in age-standardized DALY rates. According to the BAPC model’s predictive analysis, the ASDR for each stage of hepatitis B in China is projected to decline from 2021 to 2035. The ASDR for total liver diseases caused by hepatitis B is expected to decrease significantly from 775 per 100,000 population in 1990–214 per 100,000 population in 2035. The estimated ASDR for chronic hepatitis B, including cirrhosis, in China is predicted to decline from 480 per 100,000 population in 1990–97 per 100,000 population in 2035, following the same downward trend as the global burden.The DALYs for acute hepatitis B is expected to decrease from 74.5 per 100,000 population to 2.2 per 100,000 population, showing a rapid decline initially followed by a relatively stable period with a slight further decrease. The DALYs for liver cancer caused by hepatitis B is projected to decrease from 273/100,000 population to 149/100,000 population, displaying a fluctuating trend but an overall decline (Fig 6).

## Discussion

Hepatitis B is a major public health issue in both China and globally [32]. The Chinese population continues to bear a substantial hepatitis B virus (HBV) infection burden, with significant public health implications. In 2019, the global prevalence of chronic HBV infection across all age groups was estimated at 4.1% (95% uncertainty interval [UI] 3.7 to 4.5), which corresponds to 316 million (284–351) infected individuals [33]. The burden of chronic hepatitis B is highest in the Western Pacific region, followed by Africa, the Eastern Mediterranean, Southeast Asia, Europe, and the Americas [34,35]. Despite ongoing advancements in scientific research and treatment methods, hepatitis B continues to cause a substantial number of deaths annually, indicating that hepatitis B and its long-term complications remain significant health challenges worldwide.

This investigation employed comprehensive data from the Global Burden of Disease (GBD) repository to conduct a systematic assessment of temporal trends and burden patterns associated with HBV-related hepatic disorders in China from 1990**–**2021. It revealed significant differences in overall risk, disease stages, sex and age groups, and predicted the disease burden through 2035. These findings provide critical evidence-based insights for optimizing HBV prevention strategies and strengthening disease control measures in China.

This study revealed that the total number of deaths due to liver diseases caused by hepatitis B increased by 1.83% over the preceding thirty-year period, indicating a minimal increase. However, the ASMR and hepatitis B-related DALYs significantly declined. Possible causes of this phenomenon include population aging, increases in population size, and alterations in the demographic structure [36,37], which cause discrepancies between the decline in standardized indicators and the stability of absolute values. The Chinese government has implemented increasingly comprehensive strategies to prevent HBV transmission, including immunization [38], the promotion of safe injection practices, blood donation screening, and monitoring. With continuous improvements in vaccination coverage, antiviral treatments, and early diagnosis, the mortality risk and overall health burden associated with hepatitis B have been effectively controlled [39,40].

From the perspective of disease stages, the mortality rates of acute HBV infection and chronic HBV infection with or without hepatic fibrosis progression have been declining annually, reflecting the effectiveness of the current prevention and treatment measures. However, the number of deaths caused by hepatitis B-related liver cancer (which has increased by 63.14%) and the corresponding DALYs have significantly increased, and the overall burden has increased. This epidemiological pattern may be attributed to the extended latency period from chronic HBV infection to hepatocellular carcinoma development, the complexity of pathological transformation mechanisms, and population aging [41]. After HBV infection, several decades may be needed for progression to liver cancer. Therefore, the liver cancer cases observed currently may have stemmed from infections that occurred many years ago. Despite strengthened preventive measures in recent decades, the long latency of hepatocellular carcinoma has resulted in delayed observable reductions in both incidence rates and mortality outcomes. The mechanisms of HBV-related liver cancer are complex and involve viral factors, host immune responses, and environmental influences. Even with antiviral treatments, some patients may still progress to liver cancer [7,42]. This may also reflect gaps in early screening, diagnosis, and treatment of liver cancer, indicating a need for enhanced management and intervention for high-risk populations. These findings suggest that further research into the pathogenesis of hepatitis B-related liver cancer is needed to develop more effective intervention strategies.

To better respond to these challenges, some countries have implemented targeted strategies to mitigate the effects of the prolonged latency period of HBV-related liver cancer. For example, Taiwan has implemented population-based surveillance programs among HBV carriers [43], and South Korea has integrated hepatitis B monitoring into its national cancer screening framework [44,45]. These approaches, which include long-term follow-up and age-specific screening, have contributed to earlier diagnosis and improved outcomes. Such international experiences offer useful references for optimizing liver cancer prevention and early intervention efforts in China.

Building upon these strategies, the effective reduction of HBV-related mortality heavily relies on the establishment of timely diagnostic procedures and appropriate treatment regimens. The World Health Organization (WHO) recommends that by 2030, the national HBV diagnosis rate and antiviral treatment coverage should reach 90% and 80%, respectively [14]. To achieve the WHO’s goals, more effective strategies to combat HBV infection are needed. However, a significant number of infected individuals still remain unaware of their infection status and have not received standardized treatment, indicating a considerable opportunity for improvement in diagnosis coverage and treatment engagement. Therefore, both national and societal efforts are necessary to enhance these aspects [46]. 

In addition to its direct impact on mortality and disability, hepatitis B infection may contribute to a broader range of health outcomes through its association with other chronic conditions, such as chronic kidney disease, diabetes, and cardiovascular diseases [47]. Several population-based studies using data from diverse sources, including national health surveys and clinical registries, have reported potential links between chronic HBV infection and these comorbidities [48–50]. These findings underscore the importance of adopting a multidimensional perspective when assessing the full burden of hepatitis B. Future research should further explore these associations using integrated data from multiple sources to better inform clinical management and public health strategies.

Hepatitis B-related liver disease and liver cancer not only severely affect the health of patients but also place a significant burden on the national economy. A recent systematic review estimated the average annual per-patient economic burden of HBV-related diseases at approximately 92 978 RMB, with direct and hidden costs—especially for hepatocellular carcinoma—being particularly high [51]. Another study modeled the lifetime healthcare costs for the estimated 89.2 million adults living with HBV at approximately USD 1 305 billion, underscoring the massive economic impact on the health system and society [52]. Therefore, in the context of global universal health coverage, the government should actively respond to the call, increase financial investment, and develop scientifically reasonable investment plans to promote the implementation of hepatitis prevention and control measures and achieve optimal outcomes in terms of cost-effectiveness. Research shows that by 2030, if comprehensive HBV prevention and control measures are implemented in China, every dollar invested is expected to save more than 1.5 dollars, which will significantly help alleviate both the economic and social burdens [53,54]. These findings underscore the urgent need to integrate economic evaluations into HBV surveillance and public health planning to better inform resource allocation and policy decisions.

Analysis of sex and age differences revealed that hepatitis B-related mortality and disease burden are generally greater in males than in females. In 2021, the ASMR of males was significantly greater than that of females, and males bore a greater burden across all disease stages. This may be related to sex differences in the immune response, lifestyle factors (such as alcohol consumption and smoking) [55], and healthcare utilization [56]. Studies have shown that liver fibrosis and HCC progress more quickly in men and postmenopausal women, which may be partly due to a decrease in estrogen production and reduced responsiveness to estrogen [57]. These findings indicate potential sex- and age-specific variations in behavioral patterns, risk exposure profiles, and disease pathogenesis, underscoring the necessity for differentiated approaches in the development of tailored prevention and control interventions.Therefore, public health strategies should incorporate sex- and age-specific considerations, emphasizing targeted screening, prevention, and treatment programs that address the unique risk factors and disease progression patterns in different demographic groups. Tailored interventions could improve the effectiveness of hepatitis B control efforts and ultimately reduce the overall disease burden [58].

Moreover, mortality from acute hepatitis B in children under 5 years of age is notably significant, highlighting the need to further strengthen hepatitis B prevention and early intervention in children to reduce the risk in this population. According to previous studies, with the implementation of timely vaccination at birth and antiviral treatment for mothers during the perinatal period, the incidence of hepatitis B in children under five years of age has significantly decreased [59,60]. However, some children have not fully benefited from these interventions, leading to the persistence of hepatitis B surface antigen (HBsAg) infection. Therefore, to further reduce the risk of mother‒child transmission of hepatitis B, comprehensive prenatal screening for HBsAg must be performed, along with prevention of perinatal HBV infection. High-risk infants should receive hepatitis B immune globulin, antiviral treatment, or a combination of both as part of comprehensive interventions [61]. In 2017, Pan American Health Organization (PAHO) member states extended their goal of eliminating mother-to-child transmission to include hepatitis B and other infectious diseases, receiving support from the WHO Regional Offices for the Western Pacific and Southeast Asia in 2017. This strategy aims to achieve comprehensive prevention and control of mother-to-child transmitted diseases through the integrated use of immune globulin, antiviral treatment, and other measures [62].

Decomposition analysis revealed that while population growth and aging have increased the disease burden of hepatitis B, improvements in epidemiology have played a key role in reducing the overall burden. The global landscape of hepatitis B virus infection exhibits substantial heterogeneity in both geographical distribution patterns and disease burden metrics across different regions. Studies indicate that the age-standardized incidence rate (ASIR) of chronic liver diseases, including hepatitis B-related cirrhosis is increasing in low and lower-middle sociodemographic index (SDI) regions, whereas the ASIR of hepatitis B-related liver cancer is also increasing in high-SDI regions [63]. This may be attributed to differences in healthcare resources, vaccination coverage, screening strategies, and public health policies across regions. In high-income countries, despite widespread vaccination programs, the cancer risk in chronic hepatitis B patients remains high [64]. In low-income countries, limited healthcare resources may lead to delays in early diagnosis and treatment, thereby increasing the disease burden [65]. While the global hepatitis B disease burden measured by DALYs has increased, China has achieved significant reductions in HBV-related DALYs, underscoring the effectiveness of China’s comprehensive hepatitis B prevention and control strategies.

Vaccination is considered one of the most effective methods for preventing the transmission of hepatitis B. However, in regions with lower vaccination coverage, such as Africa and South Asia, the incidence of new hepatitis B infections remains high, highlighting the urgent need to improve vaccination coverage globally [66]. Since 1992, the Chinese government has included the hepatitis B vaccine in the national immunization program, and in 2002, free hepatitis B vaccination for newborns was incorporated into the national immunization plan, significantly reducing HBV infection rates [67]. However, despite a decline in overall infection rates, the burden of hepatitis B-related liver cancer continues to increase, especially among high-risk adult populations, such as unvaccinated adults, healthcare workers, and family members of HBV carriers. In these groups, vaccination rates remain low, leading to ongoing transmission of HBV in some populations [68,69]. The burden of liver cancer due to hepatitis B is heavily influenced by population aging, and future efforts should focus on disease management in the elderly population and the promotion of more effective liver cancer screening measures among older patients [70].

Projections indicate that China’s ASDR for HBV-related diseases will maintain a downward trajectory through 2035, with particularly substantial reductions anticipated in acute HBV infection and chronic HBV infection with cirrhotic complications. This reflects the positive outcomes of China’s hepatitis B prevention and control efforts in reducing the burden of acute and chronic hepatitis B. However, liver cancer, as a late complication of hepatitis B, remains a critical public health issue that needs to be addressed. Moving forward, further optimization of early screening and treatment strategies for liver cancer is essential, as are implementing tailored prevention and intervention measures for different sex and age groups.

In conclusion, this investigation demonstrated that although China has achieved a reduction in overall hepatitis B virus (HBV)-associated liver disease risk during the past thirty years, the hepatocellular carcinoma burden has persistently increased, exhibiting substantial sex- and age-specific disparities. On the basis of these findings, future efforts should focus on consolidating existing prevention and control achievements while further optimizing strategies for high-risk populations and critical disease stages, with the goal of achieving comprehensive management and precise interventions for hepatitis B.

## Study limitations

While the GBD database offers invaluable insights for analyzing hepatitis B-related epidemiological patterns in China, several methodological limitations warrant consideration. First, the original data used in the database mainly come from public statistics and sampling surveys, and data from some regions may be incomplete or inaccurately reported, which introduces a degree of uncertainty. The GBD model, when missing data are supplemented, may not fully reflect subtle epidemiological differences within different regions of China, especially regarding local healthcare resource allocation and diagnostic standards. Furthermore, there is a certain delay in data updates, which may not capture the latest progress in prevention and control. Although the GBD modeling framework has undergone extensive validation, we acknowledge that the limitations of predictive models—such as the assumptions and uncertainties inherent in the Bayesian age-period-cohort (BAPC) model—were not sufficiently addressed in the original discussion. These factors may affect the accuracy of long-term disease burden projections and should be interpreted with caution. Additionally, emerging treatments and vaccination programs could significantly impact future epidemiological trends of hepatitis B; however, these dynamic changes were not fully incorporated into the model predictions. Future studies should consider these factors to improve forecast reliability. Moreover, as the GBD 2021 database does not provide data on several important dimensions—including patient-reported outcomes (PROs), quality of life, and healthcare utilization—our study was unable to assess these aspects of the burden of hepatitis B. Future research should integrate regional cohort studies and clinical data to further investigate the specific factors influencing disease progression and management strategies.

## Supporting information

S1 Table Numbers of deaths and disability-adjusted life years (DALYs) by disease stage in China from 1990 to 2021. (XLSX)

S2 Table Age-standardized mortality rates (ASMR), age-standardized disability-adjusted life year rates (ASDR), and their 95% uncertainty intervals (UIs) by disease stage in China from 1990 to 2021. (XLSX)

S3 Table Numbers of deaths, mortality rates, and 95% confidence intervals by age group, disease stage, and sex in China in 2021. (XLSX)

S4 Table Disability-adjusted life years (DALYs), age-standardized DALY rates (ASDR), and 95% confidence intervals by age group, disease stage, and sex in China in 2021. (XLSX)

S5 Table Mortality rates and disability-adjusted life years (DALYs) rates by disease stage, sex, and age groups (<35, 35–49, 50–69, ≥ 70, and all ages) in China from 1990 to 2021. (XLSX)

S6 Table Decomposition analysis of changes in hepatitis B virus (HBV) burden by region (China vs. Global), sex, and disease stage, 1990–2021. (XLSX)

S7 Table BAPC model projections of mortality and DALYs rates for each disease stage in China from 2022 to 2035, with 50%, 60%, 70%, 80%, and 95% uncertainty intervals. (XLSX)

S1 Fig Temporal trends of DALYs of the total burden related to hepatitis B and three disease stages for all ages and across four age groups (<35, 35–49, 50–69, and ≥70 years) from 1990 to 2021 by sex and age group in China. DALYs: disability-adjusted life-years.
(TIF)

S2 Fig This diagram summarizes the main steps of our study, including data sourcing from the GBD 2021 database, stratified processing by disease stage, age, and sex, application of EAPC and decomposition analysis, forecasting using the BAPC model, and presentation of both historical and projected hepatitis B burden in China. (TIF)
