# Supplementary figures and images for: Epidemiological trends and projections to 2035 of hepatitis B burden in China, 1990–2021
Source: PLoS One. 2025 Sep 2;20(9):e0330633. doi: 10.1371/journal.pone.0330633 (PMC12404462; doi:10.1371/journal.pone.0330633)

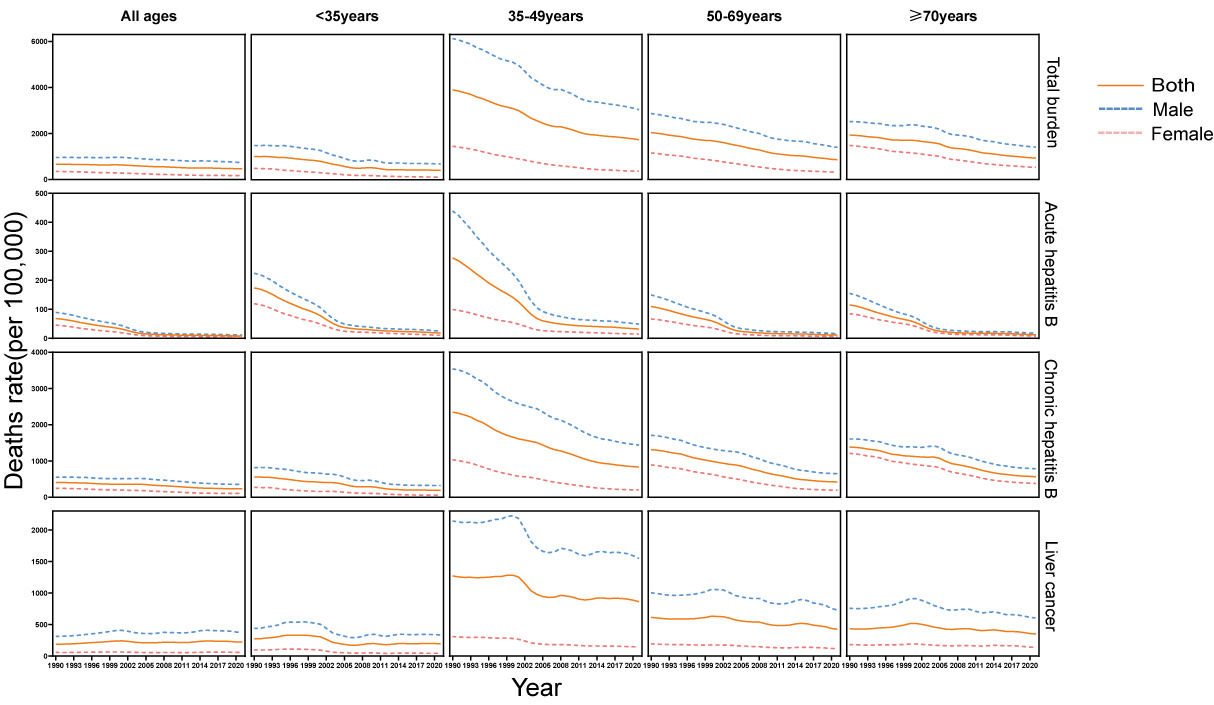

Supplement: S1 Fig — DALYs: disability-adjusted life-years. (TIF) [file pone.0330633.s008.tif]

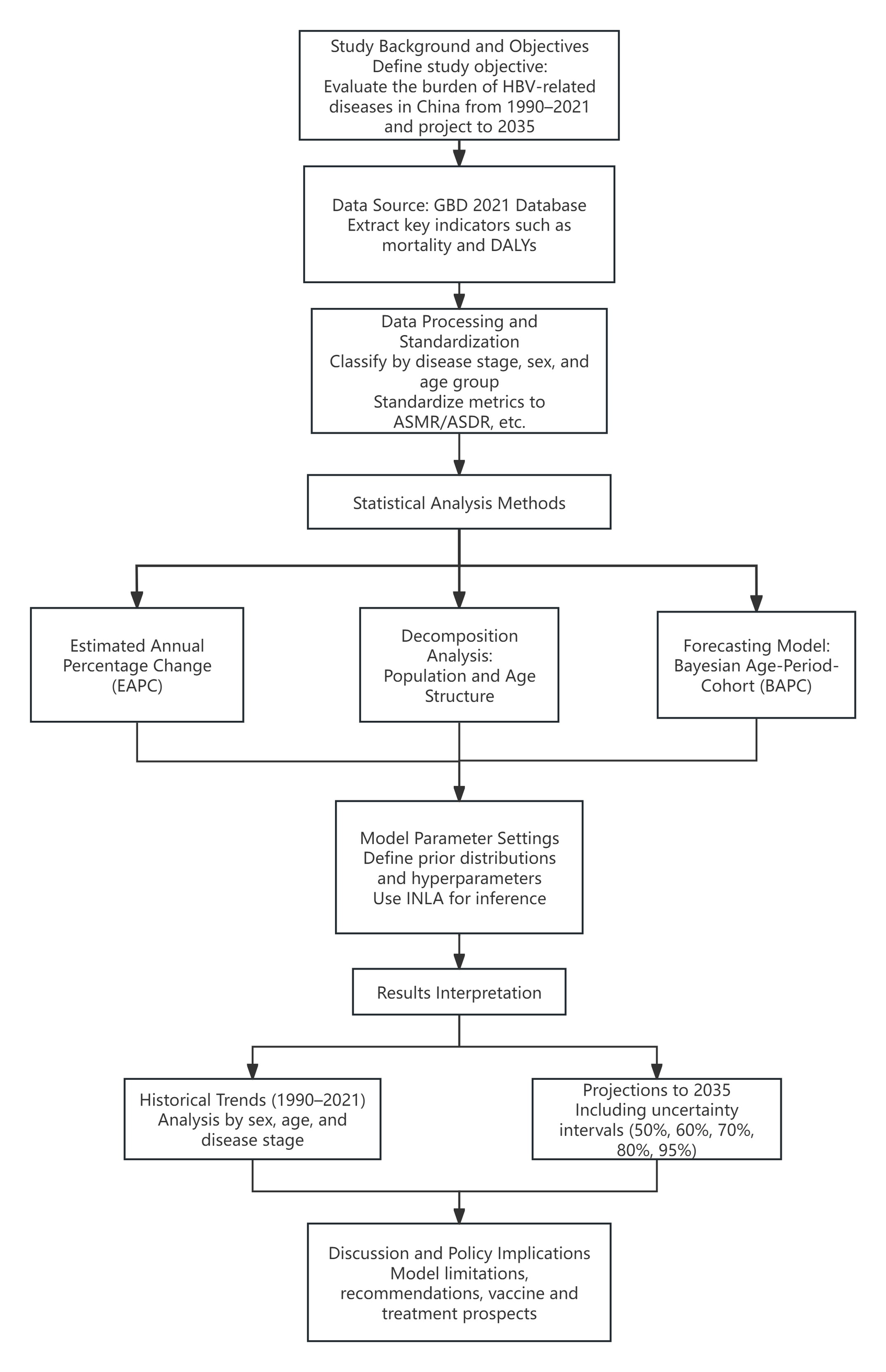

Supplement: S2 Fig — (TIF) [file pone.0330633.s009.tif]
